# Gender-differences of in vitro colonic motility after chemo- and radiotherapy in humans

**DOI:** 10.1186/s40360-018-0238-x

**Published:** 2018-08-03

**Authors:** Maria Antonietta Maselli, Antonia Ignazzi, Francesco Pezzolla, Annunziata Scirocco, Dionigi Lorusso, Fabrizio De Ponti, Carola Severi

**Affiliations:** 1 Experimental Pharmacology Laboratory, National Institute of Gastroenterology “S. de Bellis”, Research Hospital - Castellana Grotte (BA), 70013 Castellana Grotte, Italy; 2 Department of Surgery, National Institute of Gastroenterology “S. de Bellis”, Research Hospital - Castellana Grotte (BA), 70013 Castellana Grotte, Italy; 3 Department of Medical and Surgical Sciences, University of Bologna, 40126 Bologna, Italy; 4 Department of Internal Medicine and Medical Specialities, University Sapienza, 00161 Rome, Italy

**Keywords:** Chemotherapy, Radiotherapy, Capecitabine, Carbachol, Histamine, Nitrergic pathways, Motility, Human colon, Gender

## Abstract

**Background:**

The aim of the present in vitro study was to investigate, in different genders, motor responses in surgical colonic specimens from patients with rectal cancer undergoing and not undergoing chemotherapy with capecitabine and radiotherapy.

**Methods:**

This in vitro study was conducted from October 2015 to August 2017 at the Experimental Pharmacology Laboratory at the National Institute “S. de Bellis” after collecting samples at the Department of Surgery. Segments of sigmoid colon were obtained from 15 patients (Male (M)/Female (F) = 8/7; control group, CG) operated on for elective colorectal resection for rectal cancer without obstruction and 14 patients (M/F = 7/7; study group, SG) operated on for elective colorectal resection for rectal cancer who also received chemotherapy, based on capecitabine twice daily, and radiotherapy. Isometric tension was measured on colonic circular muscle strips exposed to increasing carbachol or histamine concentrations to obtain concentration-response curves. The motor responses to electrically evoked stimulation were also investigated.

**Results:**

In males, carbachol and histamine caused concentration-dependent contractions in the CG and SG. An increased sensitivity and a higher response to carbachol and histamine were observed in SG than CG (*P* < 0.01). On the contrary, in females, the response to carbachol was not significantly different in CG from the SG and the maximal responses to carbachol were greater in CG than in SG (*P* < 0.001). The same applied to histamine for half-maximal effective concentrations and maximal response in that they were not significantly different in CG from the SG. Electrically evoked contractions were significantly more pronounced in males, especially in the SG (*P* < 0.05).

**Conclusions:**

This preliminary in vitro study has shown gender differences in motor responses of colonic circular muscle strips in patients who had received chemotherapy with capecitabine and radiotherapy.

## Background

The clinical manifestations associated with exposure to chemotherapy and radiotherapy include diarrhea, abdominal cramps and associated pain and, most frequently, nausea and vomiting [1, 2]. Diarrhea involves the interaction of motor activity and epithelial transport. Mucositis is a key factor to be taken into account after chemo- and radiotherapy [3].

Some authors have shown hypercontractility in gastric fundic and duodenal muscle in rats after exposure to Fluorouracil (5-FU) [4] while others have found increased sensitivity to cholinergic stimulation in the duodenum and colon after whole-body irradiation in guinea pigs [4, 5]. This increased responsiveness after irradiation may be associated with colonic motor alterations consisting in giant contractions in dogs and intestinal migrating motor complexes disruption in rats [2, 6].

The use of chemo- and radiotherapy disrupts the mucosal barrier and represents a clinically significant risk factor for local infections and sepsis [7]. Some studies demonstrated significant enhancement in gastrointestinal muscle contractility in response to carbachol during infections and chronic inflammation [8, 9] and increased sensitivity to histamine in inflammatory conditions related to the release of leukotrienes that increase histamine receptor numbers [10]. Furthermore, the increase in gastrointestinal transit occurring during infection is mediated by endogenous tachykinins, modulated by the activation of nitric oxide (NO) biosynthesis [11]. This tachykinergic component is most probably expressed in elderly male patients per a gender-related difference in receptor density of human colon [12].

Nevertheless, this increased in sensitivity to contractile agonists is not universally observed. For instance, Sung et al. showed a significant reduction in contractility to carbachol in human stomach induced by cisplatin-based chemotherapy [13]. Besides, the response to histamine did not change in colitis in rats [14].

In 5-FU-induced oral mucositis, NO is also involved. In fact, previous reports showed that proinflammatory cytokines stimulate inducible NO synthase (iNOS)-derived NO production [7]. The NO increase is in agreement with studies reporting increased plasma nitric oxide after oral administration of anticancer drugs in rats and in disagreement with other studies reporting reduced expression of the inducible form of NOS in rats receiving cisplatin and no change in neural NO synthase (nNOS) expression in patients receiving chemotherapy [13, 15, 16].

These discrepancies in contractile response to agonists, carbachol and histamine, and NO production might be related to differences in species, intestinal tracts, duration of chemotherapy and radiotherapy or length of time after chemo-and radiotherapy as well as to gender.

Possible gender differences in the colonic response to chemotherapy and radiotherapy have not been well characterized. Therefore, the aim of the present in vitro study was to investigate, in different genders, the response to contracting agents (carbachol or histamine) and electrical stimulation in surgical colonic specimens from patients who had undergone standard chemotherapy with capecitabine and radiotherapy, compared to patients who had not undergone chemo- and radiotherapy.

## Methods

This in vitro study was conducted at the Experimental Pharmacology Laboratory of The National Institute of Gastroenterology “S. de Bellis” Castellana Grotte (BA) after collecting samples at the Department of Surgery of the same institute. The in vitro experiments were carried out from October 2015 to August 2017. Segments of sigmoid colon obtained from 15 patients (M/F = 8/7) operated on for elective colorectal resection for rectal cancer served as a control group (CG) and 14 patients (M/F = 7/7) operated on for elective colorectal resection of rectal cancer who had received neoadjuvant therapy, based on capecitabine (Xeloda®, 825 mg/m^2^, Welwyn Garden City, United Kingdom), twice daily and one dose of radiotherapy (50.4 Gy in 28 fractions of 180 cGy/die) before surgery served as a study group (SG). Patients were 15 men (8 CG, average age 73.4 ± 1.1 years; range 73–76 years and 7 SG, average age 73.3 ± 1.7 years; range 72–77 years) and 14 women (7 CG, average age 74.3 ± 1.6 years; range 72–76 years and 7 SG, average age 73.6 ± 1.3 years; range 72–78 years).

In all patients, preanaesthetic medication was intramuscular atropine (1 mg) and midazolam (5 mg). Anaesthesia was induced by propofol (2.5 mg/kg) intravenously (i.v.) and maintained with nitrous oxide/oxygen (N_2_O/O_2_) (1/2) and sevoflurane (0.5–2%). Patients received norcuronium (8 mg) i.v. during induction of anaesthesia.

Colonic strips were taken from a carcinoma-free area, 1–4 cm along the longitudinal axis.

### Motility study in vitro

We used the same methods reported before [17]. In brief, tissues were gently deprived of the mucosa and the muscle segments were cut into strips (1.0 cm × 0.3 cm) transversely (circular) to the axis of bowel. Circular muscle strips were mounted in an organ bath (10 ml) containing oxygenated Krebs solution, maintained at a temperature of 37 °C and gassed with 5% carbon dioxide (CO_2_) in O_2_. The composition of the Krebs solution was as follows: sodium chloride (NaCl) 115.5 mM; potassium chloride (KCl) 4.6 mM; calcium chloride (CaCl_2_) 2.5 mM; magnesium chloride (MgCl_2_) 2.1 mM; sodium bicarbonate (NaHCO_3_) 21.9 mM; monosodium phosphate (NaHPO_4_) 1.2 mM; and glucose 15.5 mM. Tissues were stored overnight at 4 °C prior to use. Strips were tied with a silk ligature to an isometric transducer (Force 100, N. S/N 77648, 77645, 77631, ADInstruments Ltd., Unit B, Bishops Mews, Transport Way, Oxford OX4 6HD, UNITED KINGDOM), and contractions were recorded on a PowerLab/800 (Model ML119 Serial 2265, ADInstruments Ltd., Unit B, Bishops Mews, Transport Way, Oxford OX4 6HD, UNITED KINGDOM) acquisition program. The muscle length was measured directly in the bath by using a caliper. After gentle, repeated washings following by 1-h stabilization, tissues were placed under a tension of 20–24 mN (in preliminary tests it was shown that this baseline tension produces the maximal response to 1 × 10^−6^M carbachol). After equilibration, at least two comparable responses to carbachol were recorded before tissues were exposed to increasing concentrations of carbachol, histamine or electrical field stimulation (EFS). The motor response to carbachol and histamine were investigated on circular strips obtained from male and female patients of both groups. Strips were exposed to increasing concentrations of carbachol (1 × 10^−8^M-1 × 10^−6^M, cumulatively) and histamine (1 × 10^−8^M-1 × 10^−6^M, cumulatively) in order to obtain concentration-response curves. The curves were repeated after 60 min and some washing steps.

The motor responses to EFS were investigated on circular strips obtained from male and female patients of both groups. EFS was performed with an electrical stimulator type 215/T (Hugo Sachs Elektronik, March/Freiburg, Germany) using two platinum wire electrodes (15 mm in length and 5 mm in width) placed parallel to the longitudinal axis of the strips. Electrodes were placed 4.5 mm from the strip. The duration of the train of electrical pulses was always 10 s, and stimulation was performed every 2 min (0.1–10 Hz, pulse duration 0.3 ms, supramaximal strength: 20 V) [18, 19]. The current applied across the bath was monitored and the compliance of voltage was also measured. The time interval between each train of electrical pulses was always 60 min, because preliminary experiments indicated that this allows to evoke reproducible control responses, apart from the first response, which was always discarded. Electrically evoked contractions were evaluated in the presence of atropine (2 × 10^−6^M), guanethidine (5 × 10^−6^M) and L-nitro arginine methyl ester (L-NAME 2 × 10^−4^M), an inhibitor of NO synthase. The contact time was 30 min for atropine and guanethidine and 45 min for L-NAME. The concentration of atropine was chosen on the basis of previous experiments showing that they fully blocked the contraction produced by 1 × 10^−4^M carbachol. The concentration of guanethidine was chosen on the basis of literature data [20, 21]. At the end of each experiment, strips were removed, blotted, and weighed.

### Chemicals

Carbachol chloride, histamine dihydrochloride, atropine sulphate, guanethidine sulphate, and L-nitro arginine methyl ester (L-NAME) were purchased from Sigma Chemical Co. (St Louis, Missouri, United States). Other chemicals were of the highest quality and were obtained from commercial sources.

### Statistical analysis

The results are given as means ± standard error (SE) (n indicates the number of patients). Carbachol- and histamine-induced contractions were measured as increases over baseline activity and were calculated as mN per cross sectional area (expressed in cm^2^) [18, 19, 22, 23]. We then expressed concentration-response data as a percentage of the maximal response to carbachol or histamine and calculated half-maximal effective concentration (EC_50_) values with 95% confidence intervals (CIs) from log concentration-response curves using non-linear regression analysis. Stress induced by electrical stimulation was expressed as mN per cross sectional area [18, 19, 22, 23]. We compared data sets with a paired Wilcoxon test (responses before and after treatment), unpaired Mann-Whitney U test (differences between groups), and unpaired Student’s t test, as appropriate. We used a commercial statistical package (GraphPad Prism, version 5 for Windows, GraphPad Software, San Diego, California, United States) and considered statistical significance achieved when *P* < 0.05.

## Results

### Response to carbachol and histamine

**Fig. 1:**
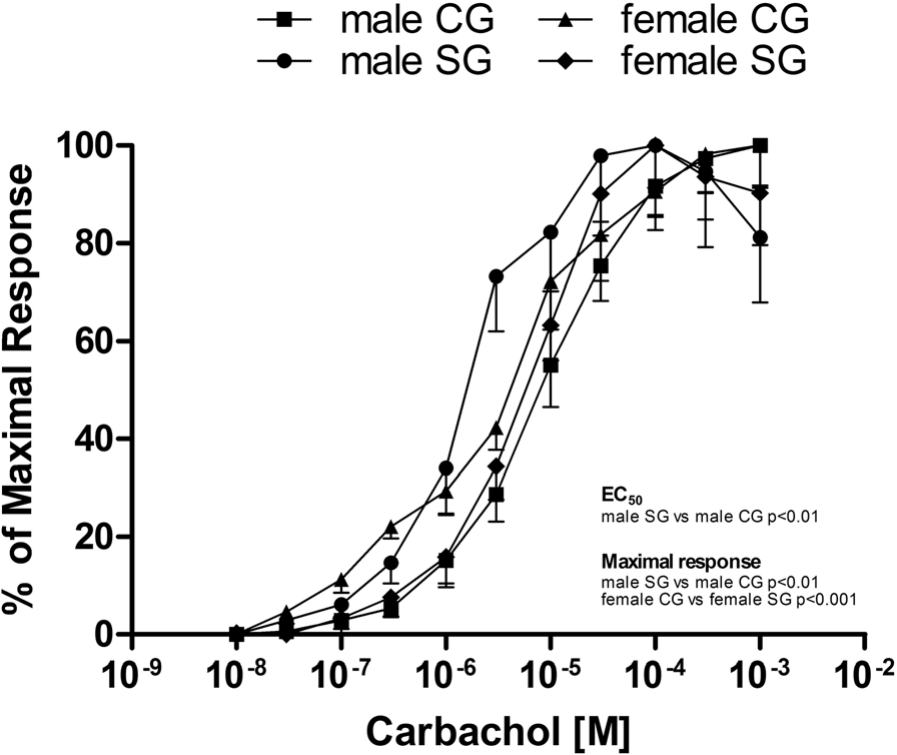
Log concentration-response curves for carbachol in male and female patients [control group (CG), study group (SG)]. All responses are expressed as a percentage of the control maximal response to carbachol. Results are means ± standard error (SE) (*n* = 15 male patients and *n* = 14 female patients). *P* < 0.01, half-maximal effective concentration (EC_50_) value in SG male patients vs CG male patients; P < 0.01, maximal response in SG male patients vs CG male patients; *P* < 0.001, maximal response in CG female patients vs SG female patients

In male patients, carbachol (1 × 10^−8^M-1 × 10^−6^M) and histamine (1 × 10^−8^M-1 × 10^−6^M) caused concentration-dependent contractions in circular muscle strips in the CG and SG (Fig. 1). EC_50_ values to carbachol were 8.54 × 10^−6^M (95% CI 4.54–16.0 × 10^−6^M) and 1.58 × 10^−6^M (95% CI 0.68–3.70 × 10^−6^M) in CG and SG respectively (Fig. 1). This response was greater in SG than in CG (*P* < 0.01). Moreover, the maximal responses to carbachol (expressed in mN/cm^2^) were significantly higher in SG male patients (4623.0 ± 894.4 mN/cm^2^ in SG vs 1621.0 ± 204.5 mN/cm^2^ in CG; P < 0.01).

**Fig. 2:**
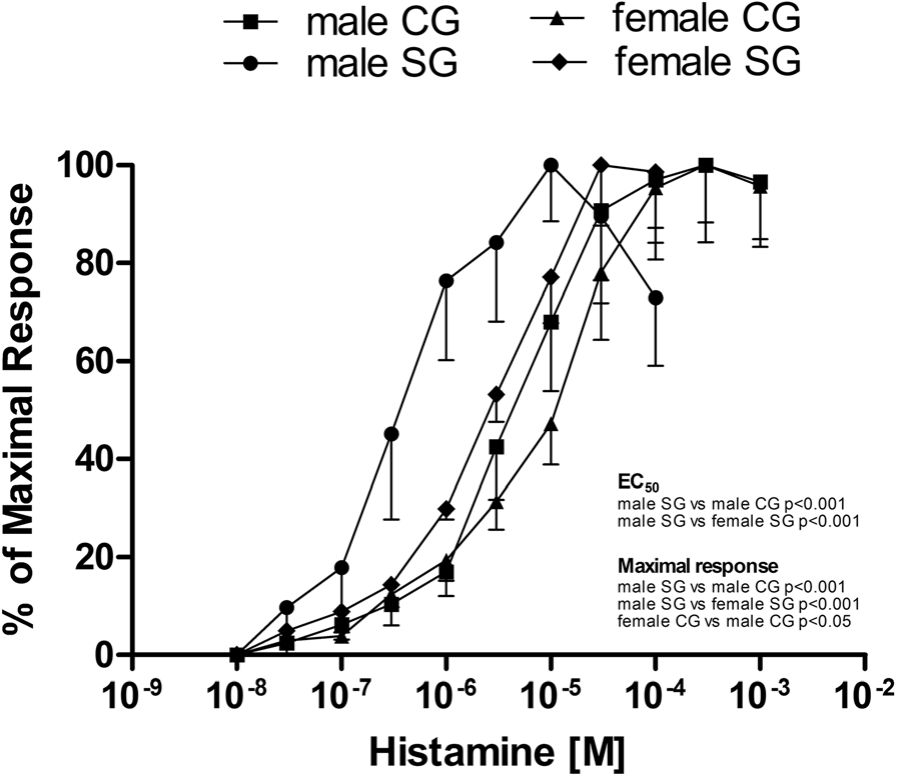
Log concentration-response curves for histamine in male and female patients [control group (CG), study group (SG)]. All responses are expressed as a percentage of the control maximal response to histamine. Results are means ± standard error (SE) (n = 15 male patients and n = 14 female patients). P < 0.001, half-maximal effective concentration (EC_50_) value in SG male patients vs CG male patients; P < 0.001, EC_50_ value in SG male patients vs SG female patients; P < 0.001, maximal response in SG male patients vs CG male patients; P < 0.001, maximal response in SG male patients vs SG female patients; *P* < 0.05, maximal response in CG female patients vs CG male patients

The same was true when we analyzed histamine-induced responses: EC_50_ values were 3.77 × 10^−6^M (95% CI 1.79–7.94 × 10^−6^M) and 0.37 × 10^−6^M (95% CI 0.09–1.42 × 10^−6^M) in CG and SG, respectively. This response was greater in SG than in CG (*P* < 0.001) (Fig. 2). Again, the maximal responses to histamine were significantly higher in SG male patients (2895.0 ± 220.0 mN/cm^2^ in SG vs 847.8 ± 123.9 mN/cm^2^ in CG; P < 0.001).

Conversely, in female patients, the response to carbachol (1 × 10^−8^M-1 × 10^−6^M) was not significantly different in the two groups, with a trend towards a reduced response in SG: EC_50_ values for carbachol were 3.84 × 10^−6^M (95% CI 1.85–7.96 × 10^−6^M) and 6.84 × 10^−6^M (95% CI 3.70–12.63 × 10^−6^M) in CG and SG, respectively (Fig. 1). The maximal responses to carbachol were significantly higher in CG female patients (2247.0 ± 186.9 mN/cm^2^ in CG vs 1354.0 ± 109.9 mN/cm^2^ in SG; *P* < 0.001).

The response to histamine was not significantly different in the two groups, with a trend towards a reduced response in CG: EC_50_ values for histamine were 7.99 × 10^−6^M (95% CI 4.27–14.97 × 10^−6^M) and 3.85 × 10^−6^M (95% CI 2.07–7.14 × 10^−6^M) in CG and SG, respectively (Fig. 2) and the maximal responses to histamine were not significantly different in the two groups (1384 ± 154.6 mN/cm^2^ in CG, 1292.0 ± 148.7 mN/cm^2^ in SG).

Moreover, analyzing groups of different gender, in SG maximal responses to carbachol and histamine were significantly more pronounced in male vs female patients (P < 0.001) and EC_50_ values to histamine were significantly different in male vs female patients (P < 0.001). In CG, the maximal response to histamine was significantly more pronounced in female vs male patients (*P* < 0.05).

### Electrically evoked contractions

In both patient groups, electrically evoked contractions in circular muscle strips were linearly related to stimulation frequency in the 0.1–10 Hz range: analyzing groups of different gender, electrically evoked contractions were significantly more pronounced in male than in female patients both in CG that in SG at all frequencies.

**Fig. 3:**
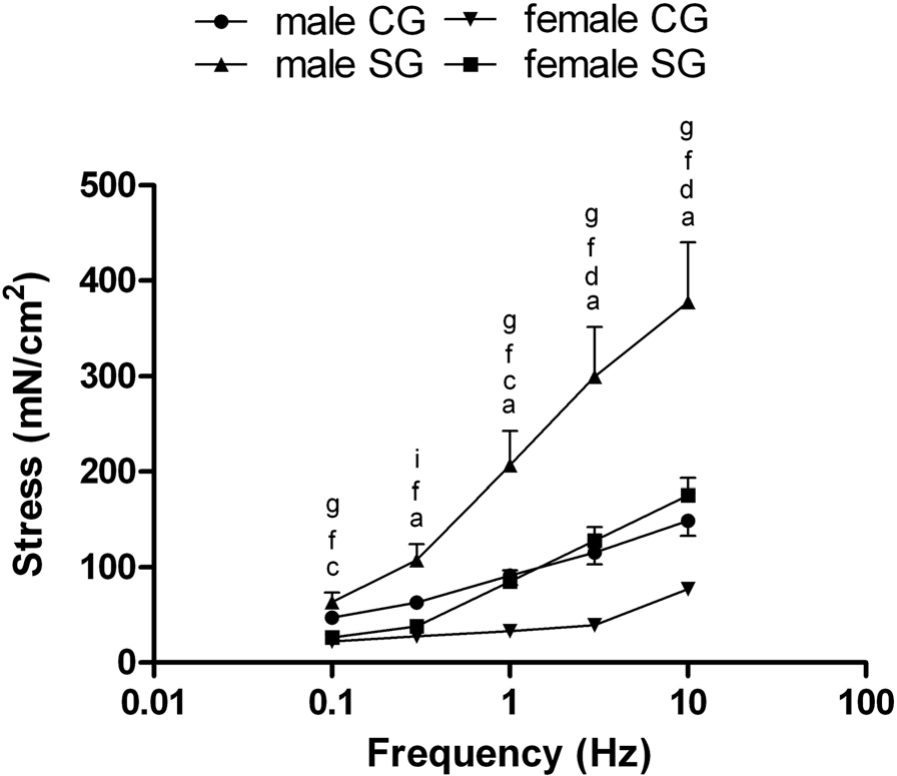
Electrically evoked contractions related to stimulation frequency (range: 0.1–10 Hz) in male and female patients [control group (CG), study group (SG)]. Results are means ± standard error (SE) (*n* = 8 male CG, *n* = 7 male SG, n = 7 female CG, n = 7 female SG). ^a^P < 0.05 and ^b^P < 0.01, CG male patients vs SG male patients; ^c^P < 0.05 and ^d^P < 0.01, CG female patients vs SG female patients; ^e^P < 0.01 and ^f^P < 0.001, CG male patients vs CG female patients; ^g^P < 0.05, ^h^P < 0.01 and ^i^P < 0.001, SG male patients vs SG female patients

The contractions in male patients were much more marked in SG than in CG in the 0.3–10 Hz frequency range (Fig. 3). The same trend was observed in female patients, where electrically evoked contractions were more marked in SG than in CG at frequency 0.1 Hz, 1–10 Hz (Fig. 3).

**Fig. 4:**
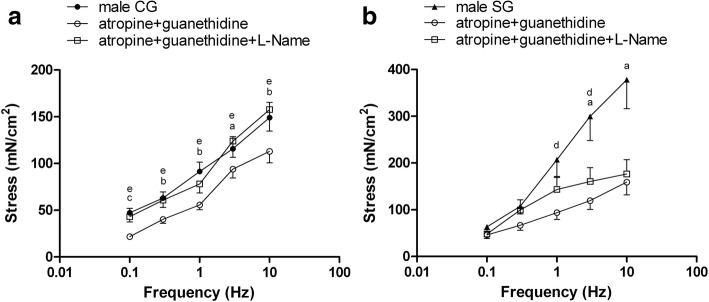
Electrically evoked contractions in control group (CG) (**a**) and study group (SG) (**b**) male patients. CG and SG experiments in the presence of atropine and guanethidine or atropine, guanethidine, and L-nitro arginine methyl ester (L-NAME). Results are means ± standard error (SE); **a**, n = 8 patients; **b**, n = 7 patients. ^a^P < 0.05, ^b^P < 0.01 and ^c^P < 0.001, atropine and guanethidine vs controls; ^d^P < 0.05 and ^e^P < 0.01, atropine and guanethidine vs atropine, guanethidine, and L-NAME

Analyzing groups of different gender, electrically evoked contractions were significantly more pronounced in male than in female patients both in CG that in SG at all frequencies. In male patients, atropine and guanethidine per se inhibited electrically evoked contractions depending on stimulation frequency. The inhibition was significant in CG at all frequencies and in SG only at 3–10 Hz. The addition of L-NAME reversed atropine and guanethidine inhibition, increasing electrically evoked responses at all frequency in CG and in SG only at 1–3 Hz (Fig. 4a-b).

**Fig. 5:**
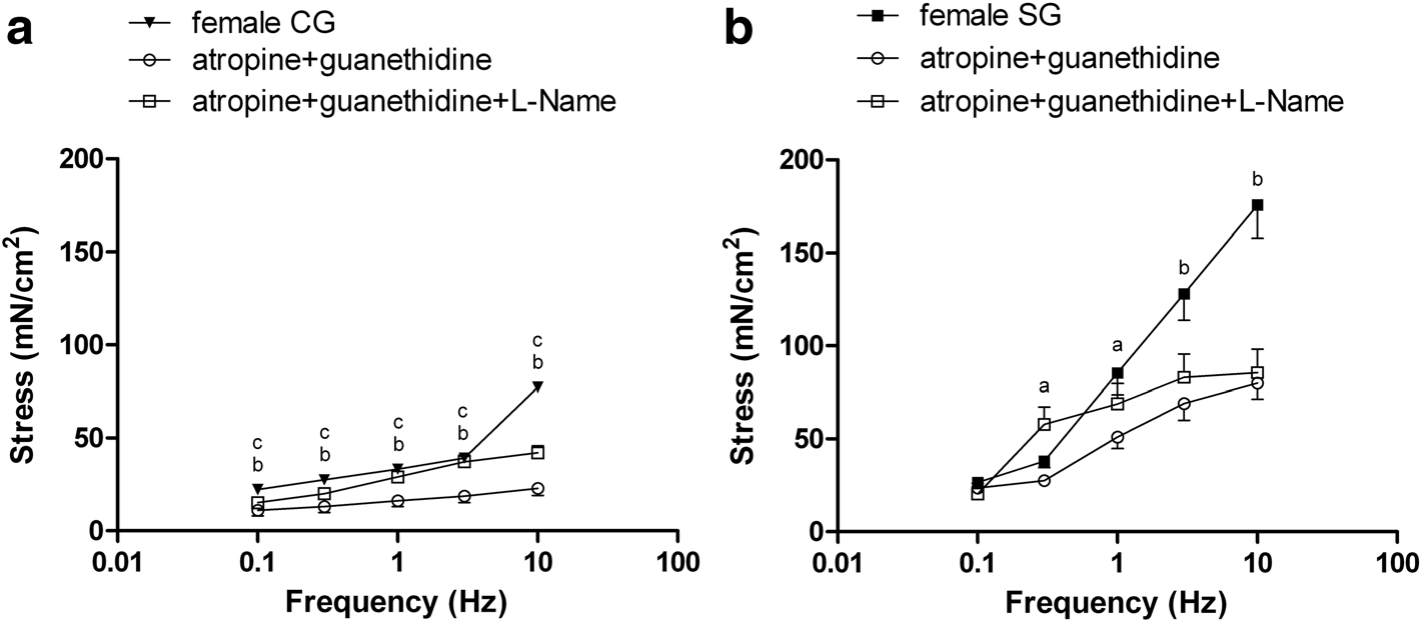
Electrically evoked contractions in control group (CG) (**a**) and study group (SG) (**b**) female patients. CG and SG experiments in the presence of atropine and guanethidine or atropine, guanethidine, and L-NAME. Results are means ± standard error (SE); **a**, n = 7 patients, **b**, n = 7 patients. ^a^P < 0.05 and ^b^P < 0.01, atropine and guanethidine vs controls; ^c^P < 0.05, atropine and guanethidine vs atropine, guanethidine, and L-NAME

In females, also atropine and guanetidine inhibited electrically evoked contractions in CG at all frequency while in SG at the frequency 0.3–10 Hz. As in male patients, the addition of L-NAME reversed atropine and guanetidine inhibition increasing electrically evoked responses in CG and in SG, but this was significant at all frequencies only in CG (Fig. 5a-b).

Notably, the effect of L-NAME was most marked in CG males and less pronounced in SG males, CG and SG females.

## Discussion

In this study, in vitro colonic motor responses to carbachol, histamine or electrical stimulation after chemo- and radiotherapy were quite different in male and female patients. Some of the observed gender differences were so remarkable that in our opinion they deserve a preliminary report here, since they are also in line with circumstantial evidence already available in the literature. However, we must acknowledge important intrinsic limitations of our study: we could analyse only a limited number of surgical specimens, which did not allow us to further investigate mechanisms underlying the gender differences. Furthermore, we cannot completely rule out the possibility that at least some of the observed differences might be due to heterogeneity of the clinical condition in individual patients.

Colonic circular muscle strips after chemo- and radiotherapy in SG were more sensitive to carbachol and histamine with respect to controls in males. Sensitivity to carbachol in these patients is in line with previous findings showing hypercontractility in gastric fundic and duodenal muscle, in both inflammatory and post-inflammatory phases of 5-FU induced intestinal mucositis in rats [4]. Other authors showed increased sensitivity to cholinergic stimulation in the duodenum and colon after whole-body irradiation in guinea pigs [5]. This increased responsiveness may be associated with colonic motor alterations consisting in giant contractions in dogs and intestinal migrating motor complexes disruption in rats [2, 6]. The increased incidence of giant contractions has been suggested to be responsible for the iatrogenic side effects such as diarrhea, abdominal cramping and vomiting [2, 6, 24]. Furthermore, other studies have demonstrated significant enhancement in gastrointestinal muscle contractility in response to carbachol during chronic inflammation and infections [8, 9]. When the mucosal barrier is damaged, the use of chemo- and radiotherapy represents a risk factor for local infections and sepsis [7].

Increased sensitivity to histamine has also been reported in inflammatory conditions related to the release of leukotrienes enhancing histamine receptor numbers [10]. Nevertheless, this increased sensitivity to contractile agonists is somewhat controversial. Sung et al. showed significantly reduced contractility to carbachol in human stomach after cisplatin-based chemotherapy [13]. Besides, the response to histamine did not change in colitis in rats [14]. These discrepancies might be related to differences in species, intestinal tracts, duration of chemotherapy or length of time after chemotherapy as well as to gender.

In the present study, the sensitivity and the maximum response to carbachol or histamine were greater in male than in female SG patients. Even considering the mean age of our patients, this different response might at least in part be ascribed to female hormones, although we cannot rule out other factors. Indeed, Kaur et al. [25] showed increased estrogen levels after surgery even in postmenopausal women. This may be an indicator than under stress (as after chemo- and radiotherapy) estrogen levels can be higher even in postmenopausal women. Therefore the different responses that we observed might be relative to an effect of female hormones still released under stress. An alternative explanation is that gender differences might be ascribed to the fact that in postmenopausal women tissues, in any case, have been exposed to estrogens in previous years, so that, even when oestrogen levels decrease, tissue responses may still differ. Interestingly, ovariectomy in adult female rabbits caused altered receptor sensitivity and increased intrinsic smooth muscle sensitivity to histamine in vascular smooth muscle. This would further explain the gender-dependent difference that we observed in females compared to males [26].

In the CG, we confirm the data previously published in our recent study on elderly female and male patients, where females were more sensitive and showed higher maximal response to carbachol [17]. These observations are in line with previous findings, one showing increased response to carbachol in animals, while the other suggested that aging induces an increase in Calcium ions (Ca^2+^) store with consequent Ca^2+^ overload, although these data do not specifically refer to gender differences [27, 28].

Also electrically evoked contractions were more marked in SG than in CG in both genders and this may be due to the formation of peroxynitrite. During severe inflammation, as observed after 5-FU treatment, a generation of reactive oxygen species (ROS) occurs. ROS directly damage cells, tissues, blood vessels and stimulate transcriptions factors, such as nuclear transcription factor-kB (NF-kB), which in turn causes up-regulation of pro-inflammatory cytokines, such as interleukin-1(IL-1), interleukin-6 (IL-6) and tumor necrosis factor alpha (TNF-alfa). Previous reports show that 5-FU induces oral mucositis causing proinflammatory cytokines that stimulate iNOS-derived NO production [7]. The reaction between NO and ROS produces peroxynitrite, a toxic compound that causes lipid peroxidation, oxidation of protein sulfhydryl groups, with tissue injury and local increase of inflammation [7, 29, 30].The increased electrically evoked contraction could also be attributed to tachykininergic components. The increase in gastrointestinal transit occurring during infection is mediated by endogenous tachykinins, which are modulated by the activation of NO biosynthesis [11]. This tachykininergic component is most probably expressed in elderly male patients. In fact, Burcher et al. showed a gender-related difference in receptor density of human colon, with binding capacity (B_max_) lower in females than in males [12]. An increase in NO is in agreement with reports of an increased plasma nitric oxide after oral administration of anticancer drugs in rats and in disagreement with other reports of a reduced expression of the inducible form of NOS in rats receiving cisplatin and of no change in neural NO synthase (nNOS) expression in patients receiving chemotherapy [13, 15, 16].

It is difficult to explain why the electrically evoked contractile response were greater in males than in females after chemo- and radiotherapy. However, even in the control group, the response to electrical stimulation is greater in males than in females, as we have already shown in previous work [17]. Besides, the response to EFS and L-NAME was lower in females than in males both in CG and in SG, possibly because elderly females are not under the influence of female hormones unlike adult females, as suggested in our previous study [17].

One study hypothesized that advanced age is associated with decreased expression of neuronal NO synthase (nNOS) and concomitant reduction in synthesis of NO in rat colon [31]. That may contribute to explain our findings in females. Other studies showed that estrogens increase endothelial NO synthase (eNOS) [32, 33], but inhibit iNOS [32]. During inflammatory reactions, NO levels, produced by iNOS, increase and this may contribute to local tissue damage [34–37]. This may explain the lack of difference in response to L-NAME group SG in both genders.

In order to explain the increased sensitivity to carbachol and decreased response to EFS and L-NAME in CG female patients we hypothesize a decreased expression of nNOS with concomitant reduction in synthesis of NO [31].

## Conclusions

This preliminary in vitro study indicates gender differences in the motor responses of colonic circular muscle strips exposed to cholinergic, histaminergic and electrical stimulation in patients who have received chemotherapy with capecitabine and radiotherapy. Further studies to elucidate the underlying mechanisms are warranted.

## Abbrevations

5-FU: Fluorouracil
EFS: Electrically evoked stimulation
eNOS: Endothelial nitric oxide synthase
IL-1: interleukin-1
IL-6: interleukin-6
iNOS: inducible nitric oxide synthase
L-NAME: L-nitro arginine methyl ester
NF-kB: nuclear transcription factor-kB
nNOS: neuronal nitric oxide synthase
NO: nitric oxide
ROS: reactive oxygen species
TNF-alfa: tumor necrosis factor alpha
M: Male
F: Female
SE: standard error
N_2_O/O_2_: nitrous oxide/oxygen
B_max_: binding capacity
CI: confidence interval
Ca^2+^: Calcium ions
i.v.: intravenous
